# Microglial exosomal miR-466i-5p induces brain injury *via* promoting hippocampal neuron apoptosis in heatstroke

**DOI:** 10.3389/fimmu.2022.968520

**Published:** 2022-10-12

**Authors:** Jie Zhu, Yahong Chen, Jingjing Ji, Longyan Wang, Guoqiang Xie, Zhen Tang, Xiangmeng Qu, Zhifeng Liu, Guangli Ren

**Affiliations:** ^1^Department of Pediatric, General Hospital of Southern Theater Command of People's Liberation Army (PLA), Guangzhou, China; ^2^Department of Critical Care Medicine, General Hospital of Southern Theater Command of PLA, Guangzhou, China; ^3^Guangdong Branch Center, National Clinical Research Center for Geriatric Diseases, Chinese PLA General Hospital, Guangzhou, China; ^4^Key Laboratory of Sensing Technology and Biomedical Instruments of Guangdong Province, School of Biomedical Engineering, Sun Yat-Sen University, Shenzhen, China

**Keywords:** heatstroke, microglia, exosome, miR-466i-5p, neuron apoptosis

## Abstract

**Background:**

Brain injury is the main cause of poor prognosis in heatstroke (HS) patients due to heat-stress-induced neuronal apoptosis. However, as a new cross-talk way among cells, whether microglial exosomal-microRNAs (miRNAs) are involved in HS-induced neuron apoptosis has not been elucidated.

**Methods:**

We established a heatstroke mouse model and a heat-stressed neuronal cellular model on HT22 cell line. Then, we detected neuron apoptosis by histopathology and flow cytometry. The microglial exosomes are isolated by standard differential ultracentrifugation and characterized. Recipient neurons are treated with the control and HS exosomes, whereas *in vivo*, the exosomes were injected into the mice tail vein. The internalization of HS microglial exosomes by neurons was tracked. Apoptosis of HT22 was evaluated by flow cytometry and Western blot *in vitro*, TUNEL assay, and immunohistochemistry *in vivo*. We screened miR-466i-5p as the mostly upregulated microRNAs in HS exosomes by high-throughput sequencing and further conducted gene ontology (GO) pathway analysis. The effect and mechanism of HS exosomal miR-466i-5p on the induction of neuron apoptosis are demonstrated by nasal delivery of miR-466i-5p antagomir *in vivo* and transfecting miR-466i-5p mimics to HT22 *in vitro*.

**Results:**

HS induced an increase in neurons apoptosis. Microglial exosomes are identified and taken up by neurons, which induced HT22 apoptosis *in vivo* and *vitro*. HS significantly changed the miRNA profiles of microglial exosomes based on high-throughput sequencing. We selected miR-466i-5p as a target, and upregulated miR-466i-5p induced neurons apoptosis *in vivo* and *vitro* experiments. The effects are exerted by targeting Bcl-2, activating caspase-3 to induce neurons apoptosis.

**Conclusions:**

We demonstrate the effect of microglial exosomal miR-466i-5p on neurons apoptosis and reveal potentially Bcl-2/caspase-3 pathway in heatstroke.

## Introduction

Heatstroke (HS) is a systemic inflammatory response associated with hyperthermia. The mortality rate of heatstroke is as high as 10%–15%, and if it develops into multiple organ dysfunction syndromes (MODSs), the mortality can reach 40% (1). The reason for the high mortality of HS is due to the poor understanding of the pathogenesis of multiorgan failure. As the central nervous system (CNS) is susceptible to hyperthermia, the brain’s metabolic rate increases, blood flow decreases, the blood–brain barrier becomes more permeable, and more inflammatory factors and pathogens enter the brain (2). Therefore, CNS is a target organ for severe HS. According to the cranial MRI results of previous HS patients, the cerebral cortex and hippocampus are the areas prone to damage. The hippocampus is an important cognitive area, its injury significantly impacts the quality of patients’ life (3). Meanwhile, in our previous studies, we found that neuronal apoptosis is an important basis for brain injury in heatstroke. We have previously focused on the damage to neurons themselves caused by heat stress but know little about the crosstalk between different types of cells in the inflammatory environment of the brain caused under HS.

Microglia are intrinsic immune cells that mediate endogenous immune responses to CNS injury and play an important role in neuroinflammation (4). The excessive immune response is an important pathogenesis of HS. Previous studies on the role of microglia in brain injury in heatstroke have mostly focused on the phenotype conversion from an anti-inflammatory to a pro-inflammatory phenotype. The interaction between microglia and neurons have not been fully elucidated. We found that microglia in HS can promote apoptosis of hippocampal neurons through a transwell co-culture system. Exosomes are important mediators of information and material exchange between cells in 30–150 nm diameter (5). Our group previously found that the expression levels of exosomal microRNA (miRNA) secreted by vascular endothelial cells and hepatocytes have differed after HS. However, the effect of microglia-derived exosomes on neurons has not been fully elucidated. This work explores further the mechanism of microglia-derived exosomal miRNA inducing hippocampal neurons apoptosis. The results of this work are expected to expand further previous understanding of the function and mechanism of microglial exosome in HS. Furthermore, the work also provides a new therapeutic strategy based on miRNAs in manipulated microglial exosomal miRNAs to improve cognitive outcome after HS.

## Materials and methods

### Animals

Male C57BL/6 (n=20, weight, 20–25 g; age, 6–8 weeks, Southern Medical University, Guangzhou, China) are maintained under controlled environmental conditions (12 h light/dark cycle, humidity, 35 ± 5%, temperature, 25˚C) at the Experimental Animal Center of General Hospital of Southern Theatre Command of PLA and are given free access to standard laboratory chow and water. The animal ethics committee of the General Hospital of Southern Theatre Command of PLA approved this study.

### HS mice model and cooling treatment

Mice in the control group are maintained at 25 ± 0.5˚C and humidity of 35 ± 5%. Mice in the HS group are placed in a pre-warmed incubator at 35.5 ± 0.5˚C and relative humidity of 60 ± 5% in the absence of food and water. The rectal core temperature (Tc) is continuously monitored with a rectal thermometer. Mice are removed from the incubator and allowed to cool at an ambient temperature of 25 ± 0.5˚C when the Tc reached 42˚C.

### Grouping and experimental procedures

The NC antagomir (Ribobio, miR03102-4-5, Guangzhou, China) and miR-466i-5p antagomir (Ribobio, miR30017325-4-5, Guangzhou, China) were explored to assess the impacts of miR-466i-5p on neuron apoptosis. Eighteen mice were randomly divided into a phosphate-buffered saline (PBS) group (n=6), NC antagomir (n=6), and miR-466i-5p antagomir group (n=6). NC antagomir group and miR-466i-5p antagomir group mice were given NC antagomir and miR-466i-5p antagomir by nasal delivery, and six control mice were given an equal volume of PBS. Mice in each group were subjected to heatstroke 24 h after nasal delivery administration and then sacrificed 24 h after HS, and the relative expression of miR-466i-5p was verified by quantitative real-time PCR (qRT-PCR). Further histopathological analysis and tests were performed.

### TUNEL assay

TUNEL assay was performed to elucidate the effect of HS microglia exosomes on neuron apoptosis *in vivo*. Twelve mice were randomly divided into two groups including control and HS exosome group. Exosomes were extracted from different groups with 1 × 10^7^ BV2 cells. Exosomes were injected through the tail vein for 3 consecutive days. Mice were sacrificed after 3 days, and further histopathological analysis and tests were performed. The TUNEL assay (TUNEL assay kit-HRP-DAB, Abcam, ab206386) was performed according to the manufacturer’ s protocol. Nuclear staining was performed with methyl green, which was in the TUNEL assay kit. The number of TUNEL-positive nuclei in the hippocampus was analyzed using ImageJ software.

### Nasal delivery of miR-466i-5p

The drug was administrated intranasally 24 h before heatstroke and then sacrificed 24 h after HS. For the treatment regimen, mice was administrated with 24 µl (1 nmol, 40 nmol/ml) of NC antagomir, miR-466i-5p antagomir, or RNase-free water and 1 µl per drop, altering drops between the left and right nose, finishing within 1 min.

### Cell culture, treatments, and CCK-8 assays

BV2 and HT22 cells were purchased from the Shanghai Institute of Cell Biology, Chinese Academy of Sciences. Cells were grown in media as recommended by the manufacturer. Dishes containing BV2 and HT22 cells were cultured in a 37°C cell incubator for 2 h as a control group, and the experimental group were maintained in 42°C for 2 h, and then rewarmed in 37°C for 12 h. Cell viability is assessed by Cell Counting Kit-8 (Dojindo, Japan) according to the manufacturer’s instructions. Cells from different treatment groups were counted, the concentration adjusted to 1 × 10^5^/ml, and seeded in a 96-well plate with 100 µl/well. Cells were seeded in triplicate for each treatment group. The 96-well plate was placed in the incubator (37°C and 5% CO_2_), and cells were cultured till the appropriate time. Ten microliters of CCK-8 solution was added in each well, and the cell culture plate was incubated for 3 h. The absorbance at 450 nm was detected using a plate reader. Blank wells (culture media and CCK) and control wells (untreated cells, culture media, and CCK) were also detected.

### Flow cytometry analysis of cell apoptosis using Annexin V-FITC/PI staining

The detection was performed according to the manual of Annexin V-FITC apoptosis detection kit (Invitrogen). About 1×10^6^ cells were collected, washed, with ice-cold PBS, and resuspended in binding buffer containing a suitable amount of Annexin V-FITC. After 10 min of incubation in the dark at room temperature, the buffer was removed by centrifugation. The cells were then resuspended in reaction buffer containing propidium iodide (PI). Flow cytometry analysis was performed immediately to detect apoptosis.

### Isolation of BV2 supernatant exosomes

The cell culture medium from control and HS BV2 cells was collected for exosome isolation. Dead cells and cell debris were eliminated with a series of centrifugations at 4°C (300×*g* for 10 min, 2,000×*g* for 20 min, and 10,000×*g* for 30 min). The supernatant was subsequently subjected to ultrafiltration through a 0.22-µm filter (Millipore, MA, USA) to remove contaminants, including apoptotic bodies, microvesicles, and cell debris. The remaining supernatant was subjected to ultracentrifugation at 100,000×*g* for 90 min at 4°C in a Beckman Coulter Optima TM L-80XP to pellet the exosomes. Next, the exosome pellets were washed with 1 ml of precooled sterile PBS, which was again ultracentrifuged at 100,000×*g* for 90 min at 4°C. Finally, the pelleted exosomes were resuspended in 30–100 µl of sterile PBS. A BCA Protein Assay kit is used to determine the protein quantity (PierceTM BCA Protein Assay kit; Thermo Fisher Scientific, MA, USA).

### Transmission electron microscopy observed exosome morphology

Five microliters of exosome suspension was added to a formvar-coated copper grid (Mecalab, QC, Canada) for 30 min, fixed in 2% paraformaldehyde for 10 min, and later stained with 2% uranyl acetate for 15 min. Exosomes were then visualized using a Philips CM10 transmission electron microscope (JEM-2100F, Netherlands).

### Nanoparticle tracking analysis

Nanoparticle tracking analysis (NTA) (NS3000, Worcestershire, UK) was employed to detect the concentration and size distribution of isolated exosomes according to the manufacturer’s instructions. Exosome samples were diluted to a concentration of 1:400 in sterile PBS, and each sample was analyzed three times with NanoSight automatic analysis settings for 60 s. A comparison of the relative protein expression profiles between the control and HS BV2-derived exosomes was performed using isobaric tags for relative and absolute quantitation technology (iTRAQ).

### Exosomal miRNA sequencing

An exosomal RNA Extraction TRIzol reagent was used to extract the RNA from exosomes. Illumina HiSeq™ 2500 was applied for sequencing, with a data volume of 20 M reads per sample. The total RNA or purified sRNA fragment of the sample was extracted. The 3′- and 5′-end junctions were ligated, reverse transcribed into cDNA, and then amplified by PCR. The target fragment library was then recovered by gum cutting, and the libraries passed quality control. Libraries were sequenced on the machine. Joints at the ends of reads and reads with fragment length <17nt and low-quality reads were removed to complete the initial filtering of data and obtain high-quality data. The clean reads were compared with the reference genome to obtain a genome-wide reads distribution map, and the clean reads were annotated with ncRNA classification. The identified miRNAs were subjected to expression calculation, miRNA expression clustering, and differentially expressed miRNA analysis between samples. For miRNAs with significant differences, the target genes of the miRNAs were further predicted, and gene ontology (GO) and Kyoto Encyclopedia of Genes and Genomes (KEGG) biological pathway enrichment analyses were performed for the target genes. We used bowtie to compare clean reads from sequencing data to the reference genome (RIBOBIO).

### Sequencing data analysis

All quantitative data were expressed as mean ± standard deviation; t-test was used for comparison between two groups, and one-way analysis of variance (ANOVA) was used for comparison of multiple groups. miRNA candidates with >2-fold up- or downregulation and p<0.05 were considered as significantly altered miRNAs. MiRNA-466i-5p was predicted as possible target molecules by TargetScan, and the predicted genes were then subjected to GO analysis, functional annotation analysis, and KEGG database analysis to determine the functional pathways involved. The 2^−ΔΔCt^ method was analyzed to explore the expression of target genes.

### miR-466i-5p mimics transfection

miR-466i-5p and NC mimics were purchased from Ribobio. To transfect HT22, miR-466i-5p mimics were diluted in Opti-MEM (Invitrogen, 31985070) to the desired final concentration. Lipofectamine 3000 (Invitrogen, L3000-001) (5 μl/well) with 125 μl Opti-MEM and 5 μl miR mimics (10 nM) with equal volume of Opti-MEM were added, and the mixture was incubated for 20 min at room temperature (RT). For six-well plate transfections, 250 μl of the miR mixture was added to each well. The medium was changed to fresh Dulbecco’s modified Eagle’s medium (DMEM) after 6 h. The relative expression of miR-466i-5p was verified by qRT-PCR after 24 h, and protein expression was verified by Western blot after 48 h.

### Real-time quantitative PCR

Total RNA was extracted by Trizol lysis (Invitrogen, CA, USA), and the concentration and quality of RNA were measured. The reaction system was configured using Takara kit TB Green Premix Ex Taq, and QuantStudio 7 Flex real-time fluorescence quantitative PCR system was used for quantitative PCR reactions to detect the expression levels of target genes. Primers were shown as follows: miR-466i-5p, F, 5′-GCGCGTGTGTGTGTGTGTG-3′ and R, 5′-AGTGCAGGGTCCGAGGTATT-3′; U6, F, 5′-CTCGCTTCGGCAGCACA-3′ and R, 5′-AACGCTTCACGAATTTGCGT-3′.

### Histopathological analysis

The brain was decapitated with a decapitating device and then immersed in 10% neutral formaldehyde to fix for 24–48 h (at 4°C). Paraffin-embedded tissues were sectioned at 3-µm thickness and stained with H&E for microscopic evaluation at a magnification of 100×, 200×, and 400×. The extent of brain injury was evaluated by two certified pathologists in a blinded manner.

### Immunohistochemistry

Paraffin sections of brain tissue specimens from each group of mice were selected for immunohistochemical staining, dewaxed in xylene, hydrated in ethanol, removed by hydrogen peroxide to remove endogenous peroxidase, then repaired using citrate solution antigen, incubated with drops of detection antibody, incubated with drops of anti-mouse secondary antibody at room temperature, added with freshly prepared DAB color development solution, nuclear stained with hematoxylin, and sealed with neutral gum after dehydration in ethanol gradient. The staining condition was observed. Under the light microscope (200×) field of view, five fields of view were randomly selected for photography. The brain tissue damage and inflammatory cell infiltration in each group were observed and compared.

### Exosome labeling

BV2 exosomes were labeled by DiD staining kit. An equal volume of dye and cells was immediately mixed and incubated for 15 min at room temperature, protecting from light, followed by termination of staining with PBS containing 1% fetal bovine serum and centrifugation at 100,000×*g* for 60 min at 4°C. The resulting precipitate was lysed in serum-free DMEM, which was the fluorescent dye. Exosomes were labeled for subsequent experiments.

### Exosome *in vitro* and *in vivo* uptake experiment

For *in vitro* uptake experiments, 10 µg of DiD-labeled exosomes was added into the recipient HT22 and incubated for 12 h. For *in vivo* uptake experiments, 40 µg of DiD-labeled exosomes was once injected into male C57/BL6 mice through the tail vein. The mice were sacrificed 6 h after the injection, and the brain tissue was frozen in −80°C and sliced. The absorption of exosomes *in vitro* and *in vivo* was observed with an Olympus FV1000 confocal scanning laser microscope. The animal ethics committee of the General Hospital of Southern Theatre Command of PLA approved this study.

### Western blot

Cell samples from each group of mice were homogenized using the method described above and then lysed using radioimmunoprecipitation assay (RIPA) lysis solution to extract the proteins, and the total proteins were separated by sodium dodecyl sulfate–polyacrylamide gel electrophoresis and transferred to nitrocellulose membranes. The tissues were incubated overnight at 4°C with the Bcl-2, caspase-3, cleaved caspase-3, and β-actin antibodies, followed by incubation with horseradish peroxidase-labeled secondary antibodies.

### Statistical analysis

For the measurement data, if the data satisfied the normal distribution and chi-square, a comparison of two sample means was performed by unpaired t-test, and a comparison of multiple sample means was performed by ANOVA to test the differences and expressed by mean ± SD (*p<0.05, **p<0.01, ***p<0.001).

## Results

### HS induces hippocampal neuron apoptosis

**Figure 1:**
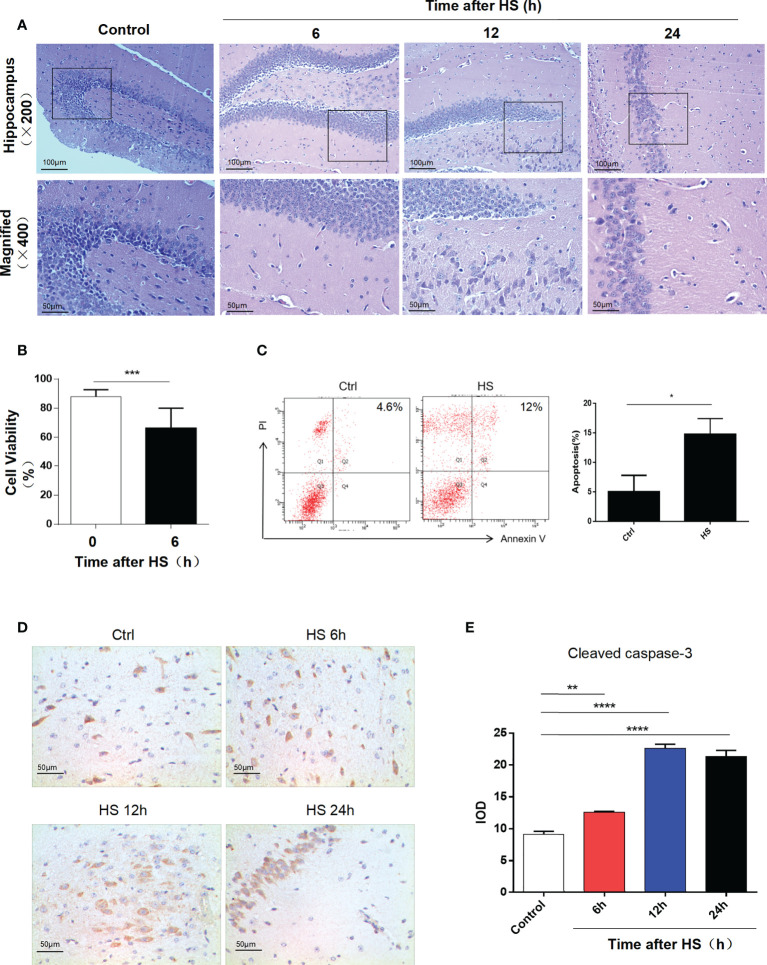
Heatstroke induces hippocampal neuron apoptosis. **(A)** Male mice were subjected to heat stress and then cooled at an ambient temperature of 25°C ± 0.5°C for 6, 12, and 24 h after the Tc reached 42°C. HE staining of the hippocampus in each group. **(B)** Cell viability assay of HT22 cell lines at 6 h after heat stress by CCK-8 assay. Data are presented as mean ± SD of three independent experiments. ***p < 0.001. **(C)** Apoptosis rate of HT22 after heat stress detected by flow cytometry. Corresponding quantifications were calculated and are shown on the right. *p < 0.05. **(D)** Cleaved caspase-3 expression was detected by immunohistochemistry in the hippocampus (400×) in control, 6, 12, and 24 h after HS. Corresponding quantifications were calculated and are shown at **(E)**. **p < 0.01, ****p < 0.0001.

After HS and cooling treatments, hematoxylin−eosin staining was performed to evaluate the degree of injury in the mouse hippocampus. The corresponding neurological injuries were graded, and the scores were assigned in a blinded manner by two certified veterinary pathologists. In 12- and 24-h groups, the neurons shrank, part of the nuclear pyknosis, and cell apoptosis appeared (**Figure 1A**). By CCK-8 assay and flow cytometry of HT22 cells for 12 h after heat stress, we found that cell viability reduced and apoptosis rate increased (**Figures 1B, C**). Caspase-3 exists as an inactive proenzyme in the cytosol and performs functions by catalyzing the C-terminal cysteine residue to specifically lyse the peptide bond following aspartic acid residues. Caspase-3 was cleaved by granzyme B at the D175 site. Then, p20 and p11 subunits were composed, which induced the activation of caspase-3. Cleaved caspase-3 can degrade intracellular structural and functional proteins and induce cell apoptosis. Immunohistochemical assessment of cleaved caspase-3 expression showed an increase in the hippocampus after heat stress and expressed the highest increase at 12 h (**Figures 1D, E**).

### Characterization of exosomes released from microglia after HS

**Figure 2:**
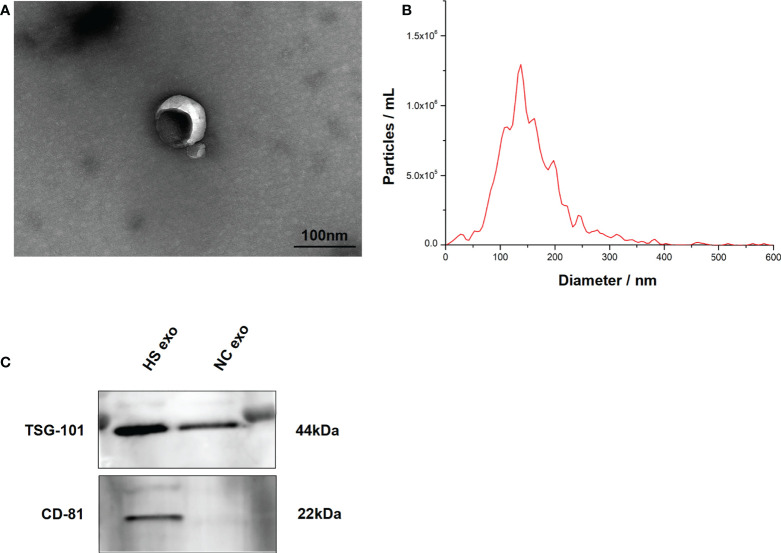
Characteristics of BV2 exosomes. **(A)** Morphology of the BV2 exosomes observed by TEM. The membrane bilayer around vesicles that are approximately 30–150 nm in diameter. Bar = 100 nm. **(B)** Size distributions of the exosomes detected by NTA showing that the diameter is predominately within the 30–150 nm range. **(C)** Representative bands of Western blotting of exosomes biomarkers with TSG101 and CD81. All experiments were replicated three times.

The exosomes secreted by control and HS BV2 cells were isolated by standard differential ultracentrifugation method. Double-layer membranous round or elliptical vesicles in diameters within 150 nm were observed under the TEM (**Figure 2A**). The size distribution of the vesicles detected by NTA was predominantly 30–150 nm, which is mostly consistent with exosomes (**Figure 2B**). Furthermore, we used Western blotting to examine the expression of the characteristic exosomal surface markers TSG101 and CD81. **Figure 2C** shows that the TSG101 and CD81 expression levels were higher in the HS group than in the control.

### Microglial exosomes induce HT22 apoptosis after HS

**Figure 3:**
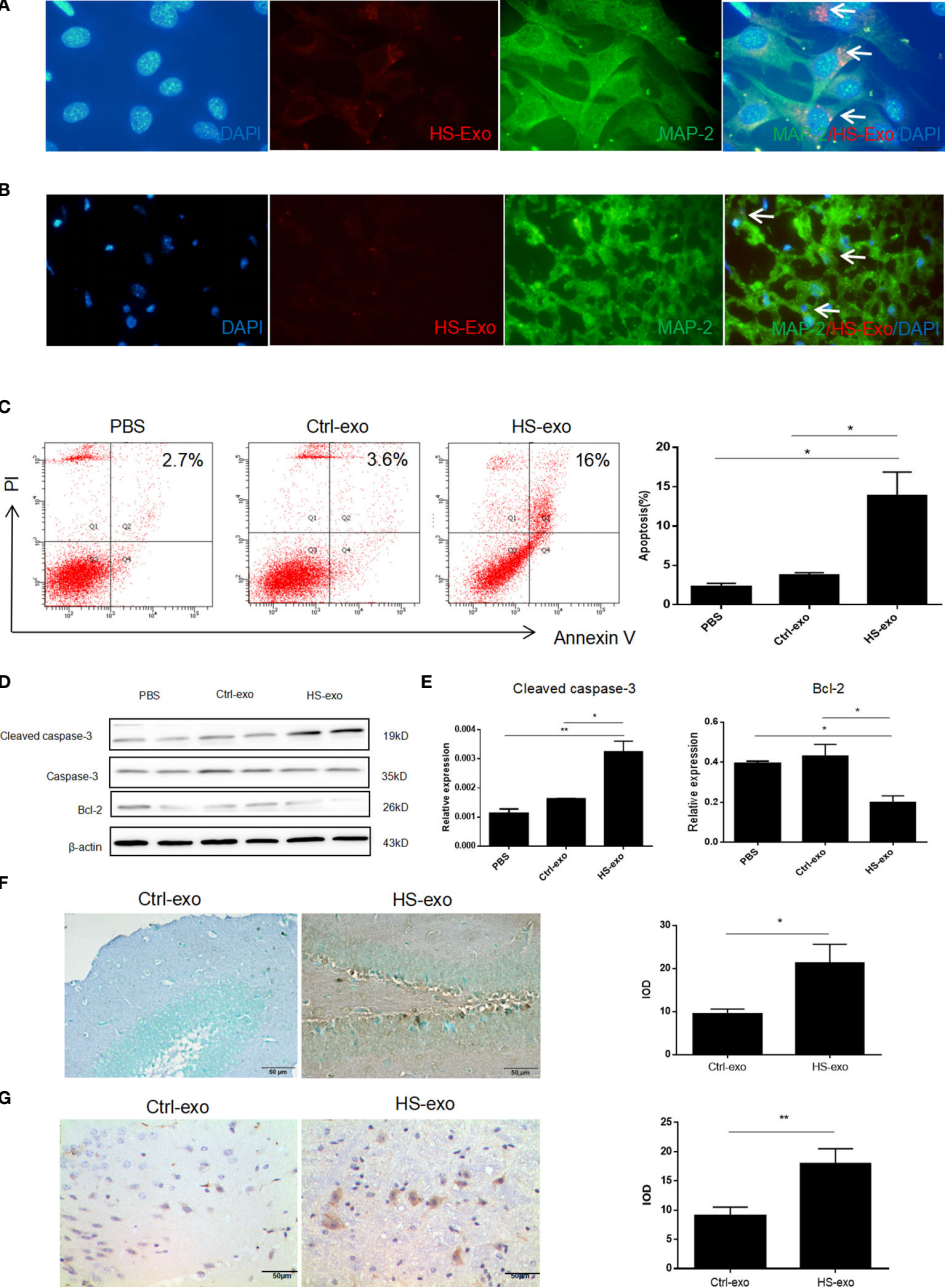
Microglial exosomes induce hippocampal neuron apoptosis in heatstroke. **(A)** Recipient HT22 were incubated with DiD-stained HS-BV2 exosomes for 12 h, and the internalization of the fluorescently labeled exosomes was visualized with a confocal scanning laser microscope. **(B)** DiD-labeled HS BV2 exosomes were injected into mice through the tail vein. Frozen sections of the hippocampus were obtained 6 h later, and the absorption of exosomes in the brain was observed under a confocal scanning laser microscope. All experiments were replicated three times. **(C)** Apoptosis rate of HT22 after co-cultured with sterilized PBS, control exosomes, and HS exosomes for 24 h detected by flow cytometry. Corresponding quantifications were calculated and are shown on the right. *p<0.05. **(D)** Expression level of Bcl-2 and cleaved caspase-3 on HT22 after co-cultured with sterilized PBS, control exosomes, and HS exosomes for 24 h was detected by Western blot. Corresponding quantifications were calculated and are shown in **(E)**. *p<0.05, **p<0.01. **(F)** Apoptosis of neurons was detected by TUNEL assay. Corresponding quantifications were calculated and are shown on the right. *p<0.05. **(G)** Cleaved caspase-3 expression was detected by immunohistochemistry in hippocampus (400×) in Ctrl-exo and HS-exo groups. Corresponding quantifications were calculated and are shown on the right. **p<0.01.

To explore the effect of microglial exosomes on HT22, we extracted exosomes from BV2 under HS to stimulate hippocampal neurons *in vitro* and *in vivo*. We examined the uptake of HS BV2 exosomes by HT22 through direct uptake experiments *in vitro*. The exosomes released from the HS BV2 were labeled with DiD and directly added to the HT22 culture medium. After 12 h of incubation, the confocal microscopy showed that red-fluorescent-labeled HS BV2 exosomes are taken up by green-fluorescent-labeled HT22 (**Figure 3A**). The *in vivo* uptake of HS exosomes by neuron was also verified. DiD-stained microglia exosomes were injected into tail vein of C57BL/6 mice. The fluorescence microscopy revealed a significant uptake of exosomes by neurons in frozen sections of the hippocampal region 6 h after injection (**Figure 3B**).

To verify the effect of microglia exosomes on hippocampal neuron function, we stimulated HT22 with extracted HS BV2 exosomes *in vitro*. The flow cytometry results showed an increased apoptosis rate of HT22 cells in the HS group (**Figure 3C**); Western blot results indicated increased expression of cleaved caspase-3 and decreased expression of anti-apoptotic protein Bcl-2 (**Figures 3D, E**). To elucidate the effect of HS microglia exosomes on neuron apoptosis *in vivo*, exosomes from control and HS groups were injected through the tail vein for 3 consecutive days. We extracted the mice brain to make paraffin sections. The TUNEL assay results showed an increased apoptosis rate of neurons in the HS-exo group (**Figure 3F**). Immunohistochemistry results also indicated increased expression of cleaved caspase-3 *in situ* (**Figure 3G**).

### Upregulation of miR-466i-5p expression in HS BV2 exosomes is associated with neuron apoptosis

**Figure 4:**
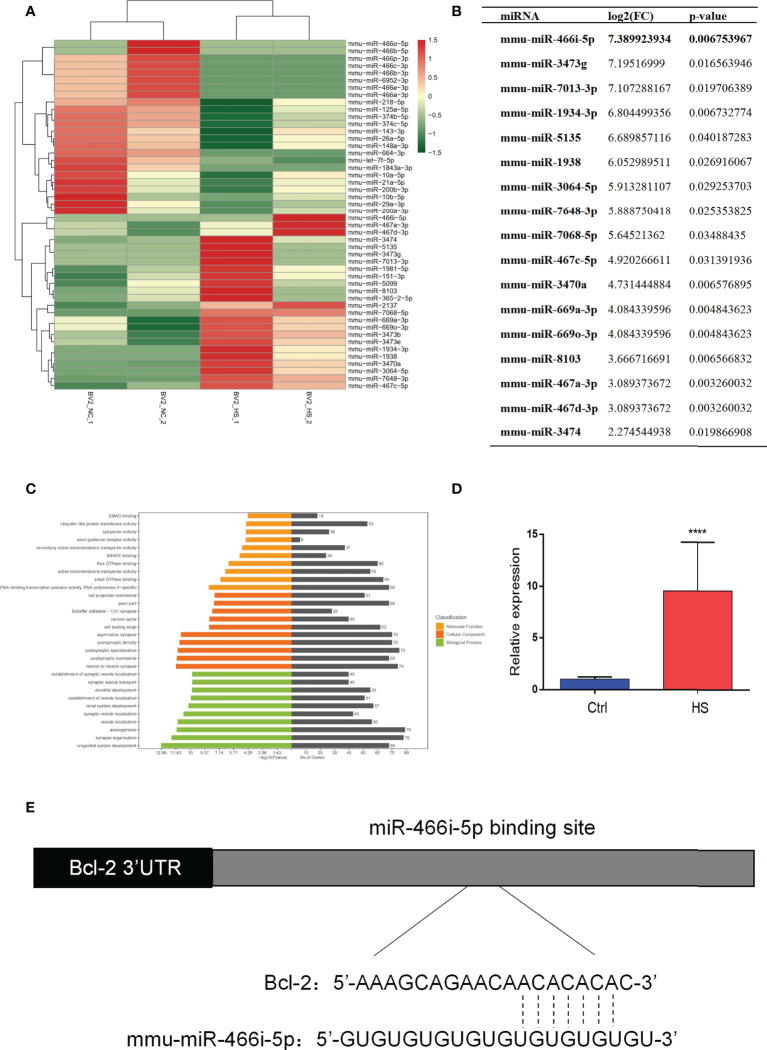
MiRNA profiling of HS BV2 exosomes and GO pathway enrichment of differentially expressed miRNA. **(A)** Heatmap of the differentially expressed miRNA components from HS EVs. The list of all 48 significantly differentially expressed miRNAs (HS-EV group/control EV group). **(B)** Fold change of miRNAs with a level change of more than 2-fold upregulation and a p-value of <0.05 was listed. miR-466i-5p is the most upregulated miRNA after HS. **(C)** Gene ontology (GO) enrichment analysis of the differentially regulated proteins. The top 30 enriched terms according to the GO functional annotation clustering of the 48 differentially expressed miRNAs in the heatstroked BV2-derived EVs. Percentages of the sequences involved are shown. **(D)** BV2 exosomal miR-466i-5p expression was detected by RT-qPCR. ****p<0.0001. **(E)** The binding sites of miR-466i-5p in the Bcl-2 3′ transcription region predicted by TargetScan (http://www.targetscan.org).

*In vitro* analysis revealed that microglia exosomes could induce apoptosis of hippocampal neurons. We harvested BV2 exosomes 6 h after heating by ultracentrifugation to explore the mechanism further. High-throughput sequencing was used to detect microRNA profiling of BV2 exosomes. **Figure 4A** shows that differences in most identified miRNAs are not significant due to the close abundance ratio of HS versus control EVs. Nevertheless, 48 miRNAs were differentially expressed (24 upregulated and 24 downregulated) in the HS group compared with the control group. Significantly elevated miRNAs in HS BV2 exosomes are shown in **Figure 4B**. MiR-466i-5p is most significantly elevated in the HS group. GO analysis helps to determine the important functions of miRNA enrichment. In this work, differentially altered miRNAs were mainly enriched on vesicle transport and synapse formation, and these functions were associated with neuron crosstalk (**Figure 4C**). Thus, BV2 exosomal miRNAs may be associated with neuron function. We further verified the expression change of miR-466i-5p in microglial exosomes using qRT-PCR. MiR-466i-5p was significantly elevated in the HS BV2 exosomes, which is consistent with high-throughput sequencing results (**Figure 4D**). Based on these findings, to further investigate the effect of microglia exosomal miR-466i-5p on neuron apoptosis, we predicted that Bcl-2 is a possible downstream target of miR-466i-5p through the TargetScan website (**Figure 4E**).

### Nasal delivery of miR-466i-5p antagomir reversed HS neuron apoptosis

**Figure 5:**
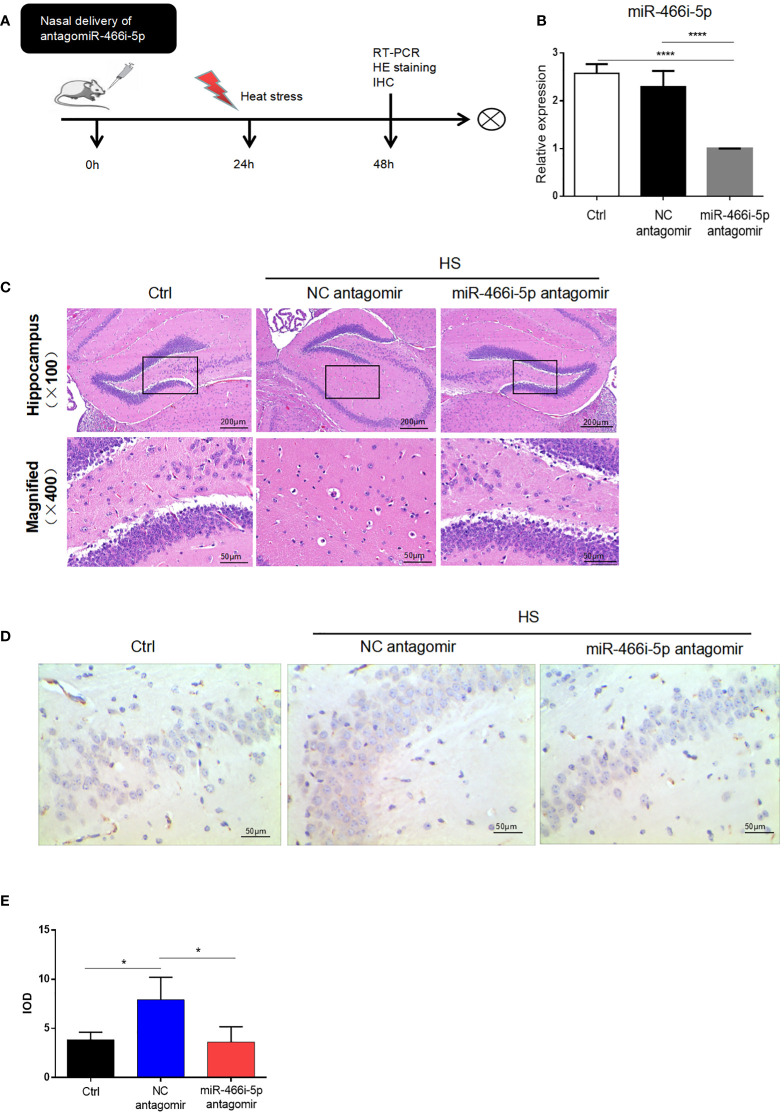
Nasal delivery of miR-466i-5p antagomir reversed HS neuron apoptosis. **(A)** The specific intervention and general process after intervention. **(B)** The relative expression of miR-466i-5p in the hippocampus at 24 h was detected by RT-qPCR. ****p < 0.0001. **(C)** HE staining of the hippocampus after nasal delivery of PBS, NC antagomir, and miR-466i-5p antagomir. **(D)** Cleaved caspase-3 expression was detected by immunohistochemistry in the hippocampus (400×). Corresponding quantifications were calculated and are shown in panel **(E)**. *p < 0.05.

To explore the function of miR-466i-5p and its effect on neuron apoptosis, miR-466i-5p antagomir was delivered into the hippocampus of C57BL/6 mice through nasal delivery. The specific intervention and the postintervention process are briefly shown in **Figure 5A**. As shown in **Figure 5B**, the result of qRT-PCR indicated that miR-466i-5p antagomir significantly reduced the relative expression of miR-466i-5p in the hippocampus, reaching a −2.296-fold at 24 h after delivery compared to that in the NC antagomir group. Hematoxylin−eosin staining was performed to evaluate the degree of injury in the mouse hippocampus. The corresponding neurological injuries were graded, and the scores were assigned in a blinded manner by two certified veterinary pathologists. In the delivery of NC antagomir group, the neurons shrank, part of the nuclear pyknosis, and cell apoptosis appeared, whereas the delivery of miR-466i-5p antagomir can reverse neuron apoptosis (**Figure 5C**). Immunohistochemical assessment of cleaved caspase-3 expression showed an increase in the hippocampus after heat stress and decrease after delivery of miR-466i-5p antagomir (**Figures 5D, E**).

### HS BV2 exosomes induce HT22 apoptosis through shuttling miR-466i-5p *in vitro*

**Figure 6:**
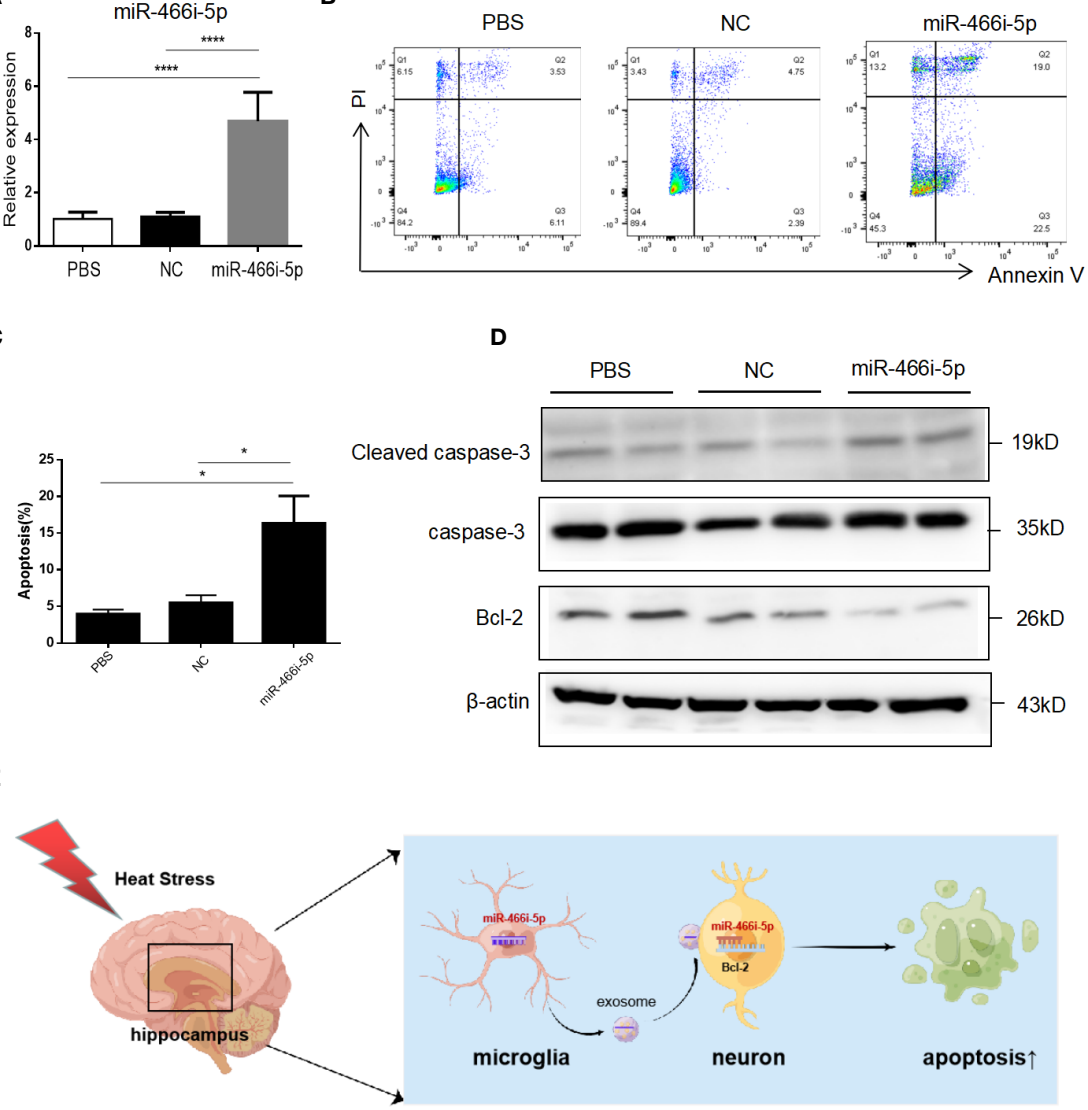
BV2 exosomal miR-466i-5p induces HT22 apoptosis *via* Bcl-2/caspase-3 pathway. **(A)** Overexpression after transfection of miR-466i-5p mimics in HT22 was detected by RT-qPCR. ****p<0.0001 **(B)** Apoptosis rate of HT22 after transfection of miR-466i-5p mimics. Corresponding quantifications were calculated and are shown in **(C)**. *p < 0.05. **(D)** Expression level of Bcl-2 and cleaved caspase-3 on HT22 after transfection of miR-466i-5p mimics. **(E)** Diagram of HS BV2 exosomal miR-466i-5p induction of HT22 apoptosis (Figdraw:https://www.figdraw.com/static/index.html#/. ID : OYSOU48a8a).

To investigate the function of microglial exosomal miR-466i-5p on neuron apoptosis, miR-466i-5p mimics were transfected into a cultured HT22 cell line to acquire upregulated miR-466i-5p and confirmed that the transfection could increase miR-466i-5p levels in HT22 (**Figure 6A**). The impact of upregulated miR-466i-5p on neuron apoptosis was detected by flow cytometry. We found that overexpression of miR-466i-5p in HT22 cells induced HT22 apoptosis (**Figures 6B, C**), and Western blot results suggested that this effect may be mediated by the Bcl-2/caspase-3 pathway (**Figure 6D**).

## Discussion

In this work, we conducted high-throughput sequencing to screen differential expression of exosomal miRNAs released from HS-induced BV2 and explored the mechanisms involved in the induction of severe HS brain injury. From the results of this work, we can conclude that hippocampus neurons can take up HS microglial exosomes *in vitro* and *vivo*. Then, absorbed exosomal miR-466i-5p can induce neuron apoptosis *via* the Bcl-2/caspase-3 pathway.

HS is a systemic inflammatory response related to heat (6), which can cause multiple organ dysfunction and failure (7). At present, there is still no effective method for the treatment of heatstroke (8). The brain is sensitive to heat, and about 80.3%–100% of patients with HS have central nervous system damage. For surviving patients, neurological sequelae are also an important factor in the quality of life. In our previous studies, we found that neuronal apoptosis is an important basis for brain injury in heat stroke (9, 10). However, due to the particular intracranial structure and complex interactions between cells, the pathophysiological mechanism of brain injury in the HS is still unclear, which is a significant difficulty in treating heatstroke.

Microglia are a kind of resident macrophages in CNS that regulate brain development, neural networks, and injury repair and are distinct from other tissue-resident macrophages due to their unique homeostatic phenotype and regulation by the CNS microenvironment (11, 12). Exosomes are a newly discovered cargo of intercellular communication (13). They transport various signaling molecules between adjacent or distant cells, including protein, miRNA, and lncRNA, thereby participating in the functional regulation of pathological processes (14). Microglia can also interact with neurons and other glial cells by releasing exosomes, which contain a variety of substances, among which miRNAs are the most common (15). Previous studies have shown that microglial exosomal miR-124 can downregulate USP14 to reduce neurodegeneration and promote synaptic growth in mice after traumatic brain injury through targeting Rela/ApoE signaling pathway (16). In addition, microglia exosomes are also involved in neurotransmitter transmission (17), synaptic growth (18), and angiogenesis (19) and play an important role in CNS injury (20) (21). The effects of microglia exosomes on neuronal function in HS have not been investigated.

Our study found increased apoptosis of hippocampal neurons under HS and further explored that microglia promoted apoptosis of hippocampal neurons through a cell co-culture system. To further investigate whether microglia promote apoptosis of hippocampal neurons by secreting exosomes, we extracted BV2 culture supernatant exosomes under heat stress and characterized them. Then, we explored the uptake of microglia exosomes by hippocampal neurons through *in vivo* and *in vitro* experiments. We stimulated the hippocampal neurons HT22 with extracted exosomes *in vitro.* Then we detected the apoptosis and expression of apoptosis-related pathway proteins Bcl-2 and caspase-3 of HT22. TUNEL assay and immunohistochemistry were performed to evaluate apoptosis of neurons in the hippocampus after injecting microglial exosomes through the tail vein. The analysis of protein or genetic contents differently expressed can provide insights into the association of exosomes with the disease and can be used to screen for novel biomarkers for the diagnosis and prognosis of disease. Our previous work revealed that exosomal miRNAs released by vascular endothelial cell (22) and hepatocyte (23) appear differentially expressed under heat stress, and hepatocyte exosomes can promote liver injury through NOD-like receptor pathways. In addition, circulating exosomal miRNAs were altered in peripheral blood of heatstroke patients, and bioinformatics analysis correlated with inflammatory response and coagulation function (24). It indicates that exosomal miRNAs play an important role in multiorgan injury in HS. Previous studies on the role of microglia in brain injury in heatstroke have mostly focused on the phenotype and MSC therapy (25), and dexmedetomidine (26) may alleviate neuroinflammation caused by heat stress by promoting the conversion of microglia from a pro-inflammatory to an anti-inflammatory phenotype. However, there are no studies on the role of microglial exosomes.

We screened miRNA-466i-5p as the most upregulated miRNA of BV2 exosomes under heatstroke by high-throughput sequencing and validated its expression by real-time quantitative PCR *in vitro*. Bioinformatics analysis revealed that the altered miRNAs might be related to neuronal function. However, the mechanism of induction of neuron apoptosis is still unclear. We predicted that Bcl-2 is a possible downstream target of miR-466i-5p through the TargetScan website. Then, we conducted mouse model and found a decrease in neuron apoptosis after nasal delivery of miR-466i-5p antagomir *in vivo*. To verify the result, we transfected miR-466i-5p mimics to HT22. We found an increased apoptosis rate in hippocampal neurons by transfecting miR-466i-5p mimics to HT22 *in vitro*, and the Western blotting results indicated that this may occur through the Bcl-2/caspase-3 pathway.

In this work, we found that microglia exosomes induced hippocampal neurons apoptosis. Therefore, we propose that HS microglial exosomes promote brain injury, possibly through exosomal miR-466i-5p binding to Bcl-2 to induce neuron apoptosis. However, our study still has some limitations that need to be addressed. First, we have not been able to clarify the distribution of miR-466i-5p *in vivo* and to confirm whether it is only expressed and upregulated in the microglia in the hippocampal tissue after exposure to HS. More detailed assessments are needed including *in situ* hybridization. Second, although we observed the pro-apoptotic effect of microglia exosomal miR-466i-5p on hippocampal neurons *in vitro*, whether exosomal miR-466i-5p or miR-466i-5p only have the pro-apoptotic effect needs to be elucidated *in vivo*. Third, Bcl-2 was predicted as a possible downstream target gene of miR-466i-5p by the Targetscan database, but we did not confirm their direct combined effect. In addition, Bcl-2 signaling pathway validation *in vitro* and *in vivo* needed to be elucidated. In view of future clinical applications, the function and mechanism of microglial exosome under heatstroke deserve future comprehensive and thorough exploration.

## Conclusions

In conclusion, this study preliminarily confirms that exosomal miR-466i-5p released by HS-induced microglia are involved in HS hippocampal neuron apoptosis and may regulate it through the Bcl-2/caspase-3 pathway. This study opens up a new perspective on the function of microglial exosomes in severe HS. It provides a basis for the subsequent verification of the biological functions of microglial exosomes and exosomal miR-466i-5p in HS microglial exosomes and provides potential intervention targets.

## Data availability statement

The datasets presented in this study can be found in online repositories. The name of the repository and accession number can be found below: National Center for Biotechnology information (NCBI) Gene Expression omnibus (GEO), https://www.ncbi.nlm.nih.gov/geo/, GSE210897.

## Ethics statement

The animal study was reviewed and approved by the Research Ethics Commission of General Hospital of Southern Theater Command of PLA and the requirement for informed consent was waived by the Ethics Commission.

## Author contributions

Study concept and design (JZ, GR, ZL, and XQ), *in vivo* experiment (JZ, YC, and LW), *in vitro* experiment (JZ, YC, and JJ), data collecting (JZ, YC, JJ, GX, and ZT), statistical analysis (GR, ZL, XQ, and JZ), and manuscript drafting (JZ, GR, ZL, and XQ). All authors contributed to the article and approved the submitted version.

## Funding

This work was supported by the grants from the PLA Logistics Research Project of China (CLB20J032, 18CXZ030 and 2022-JCJQ-ZD-097-12) and Natural Science Foundation of Guangdong Province (2021A1515010170).

## Conflict of interest

The authors declare that the research was conducted in the absence of any commercial or financial relationships that could be construed as a potential conflict of interest.

## Publisher’s note

All claims expressed in this article are solely those of the authors and do not necessarily represent those of their affiliated organizations, or those of the publisher, the editors and the reviewers. Any product that may be evaluated in this article, or claim that may be made by its manufacturer, is not guaranteed or endorsed by the publisher.
